# Physicochemical Properties and Antioxidant Activity of Portuguese Craft Beers and Raw Materials

**DOI:** 10.3390/molecules27228007

**Published:** 2022-11-18

**Authors:** Sara Silva, Ana Isabel Oliveira, Agostinho Cruz, Rita Ferraz Oliveira, Rubim Almeida, Cláudia Pinho

**Affiliations:** 1 Escola Superior de Saúde (ESS), Instituto Politécnico do Porto (IPP), 4200-072 Porto, Portugal; 2 Centro de Investigação em Saúde e Ambiente (CISA), Escola Superior de Saúde (ESS), Instituto Politécnico do Porto (IPP), 4200-072 Porto, Portugal; 3 CIBIO, Research Center in Biodiversity and Genetic Resources, InBIO-Associate Laboratory, University of Porto, 4485-661 Vairão, Portugal; 4 Department of Biology, Faculty of Sciences, University of Porto, 4169-007 Porto, Portugal; 5 PO Herbarium, MHNC-UP—Museum of Natural History and Science of the University of Porto, 4099-002 Porto, Portugal

**Keywords:** craft beer, hops, malt, physicochemical properties, antioxidant activity, phenolic compounds

## Abstract

There is an increase in the popularity of craft beer, which is produced by small, independent, and traditional breweries. Since craft beer popularity is rising in Portugal this research focused on assessing physicochemical parameters, total phenolic content (TPC) and the antioxidant capacity of Portuguese craft beers and raw materials used in beer production. In this experimental study, 19 beer samples were analyzed. Parameters such as pH, Total Acidity, Reducing Sugar Content and TPC were evaluated. For the determination of antioxidant activity, DPPH scavenging activity and metal chelating activity (MCA) were analyzed in all samples. Craft beers demonstrated a high phenolic content (ranging from 343.78 mg GAE/L to 2172.49 mg GAE/L), significantly different from industrial beers. Craft beers demonstrated a higher inhibition of DPPH radicals and higher MCA than the raw materials. DPPH inhibition ranged from 36.5% to 96.0% for malt and 64.7% to 79.6% in hops samples. MCA also varied between the different samples, with results of 12.0% to 24.8% in malt samples and 3.8% to 23.5% in hops. Raw materials can potentially influence the antioxidant activity of the resulting beer. Positive correlations between TPC and physicochemical properties can be useful to help consumers choose beers with added value for health.

## 1. Introduction

Beer is one of the most consumed alcoholic beverages in the world, and the second most consumed in Portugal. Over the years beer drinking behavior has changed, with an increase demand for craft beers [1,2]. In Portugal, there are about 640 thousand consumers of craft beer and about 115 microbreweries [1,3]. Craft beer is produced in small, traditional, and independent breweries, with an annual production of 6 million barrels or less, and a focus on organoleptic characteristics, particularly in flavor. Moreover, less than 25% of the brewery can be owned or controlled by a beverage alcohol industry member [4]. Another requirement to define a craft beer is that alcohol content must come from beer made with traditional or innovative ingredients and fermented using yeast [5]. 

Beer, made from four initial major ingredients (water, malt, hops, and yeast), has a higher nutritional value when compared to other alcoholic beverages, due to numerous compounds originated from the brewing materials and the malting and fermentation processes [6]. The main component is water (approximately 90%), followed by alcohols resulting from the fermentation process (3.5–10%), carbohydrates (1–6% *w/v*), and minerals. It also contains carbon dioxide, organic acids, inorganic salts, nitrogen, higher alcohols, aldehydes, esters, sulphur compounds, hop derivates and B complex vitamins. The main inorganic salts include calcium, magnesium, sodium, potassium, sulphate, chloride, phosphate, carbonate, and nitrate [7]. Yeast is responsible for the fermentation process and plays a fundamental role in the synthesis and formation of several compounds found in beers. There are two main yeast strains used in brewing: *Saccharomyces cerevisiae* and *S. pastorianus*, originating ale or lager beer styles, respectively. During fermentation, yeast metabolizes the carbohydrates, converting them to ethanol and carbon dioxide [8,9].

For malting, the most used cereal is barley; however, wheat, sorghum or rye can also be used. Malt is a source of carbohydrates, amino acids, proteins, and vitamins in beer. Malt also has in its composition several compounds with antioxidant activity, namely phenolic compounds and melanoidins [10,11]. There are base malts and specialty malts. Specialty malts can be classified as light-coloured, caramel (crystal) or dry roasted malts [12]. The use of cheaper raw materials can lead to industrial beers having a lower concentration of phenolic compounds, when compared to craft beers [13]. Malt and hops are the main source of phenolic compounds in beer. Hops are responsible for about 30% of the total polyphenols present in beer, although a proportion 100 times lower than malt is used [14]. The malt contributes about 70 to 80% of the total polyphenol content in beer [15]. The most reported phenolic compounds include flavonoids (e.g., flavanols, flavones), hydroxycoumarins, phenolic acids (e.g., 4-hydroxyphenylacetic, vanillic, caffeic, syringic, p-coumaric, ferulic, and synaptic acids), tannins, proanthocyanidins, and amino phenolic compounds, all of which have been related to different biological effects [13]. Polyphenols act as antioxidants, preventing oxidative degradation of beer and providing potential health benefits to consumers [16]. The phenolic compounds already identified in hops show strong antioxidant, estrogenic, anti-inflammatory, and anti-carcinogenic activities [16,17,18]. 

There are many beer styles and types, depending on the brewing process and selection of raw materials. Parameters such as sweetness, bitterness, color, alcohol volume and health attributes influence the consumer choice [19]. Craft brewers aim to combine tradition with innovation, authenticity, and product quality, making it more attractive to consumers [20]. Innovations in craft beer are related to aspects such as ingredients, alcohol content, aging, and packaging. Craft beers are often not filtered/not pasteurized, which make these beverages richer in health compounds but with a reduced shelf life [21]. Some parameters are related to the quality of craft beer such as pH, total acidity, reducing sugars and total phenolic content. Normally, beer pH ranges from 3.9 to 4.5 influencing its ageing and stability by affecting resistance to microbial spoilage, colloidal, foam and flavor stability and palate smoothness and drinkability [8]. Tracking the carbohydrate content in beer is important for the development of new flavors and selection of raw materials [10]. Measuring reducing sugars is also important to determine the shelf life of beer [22,23].

The determination of antioxidant capacity in marketed craft beers is of great interest to the public and producers because it is not common to relate an alcoholic beverage to health benefits. Due to the complex composition of beer, the study of each antioxidant compound is also expensive and inefficient, and the possible synergistic interactions between antioxidant compounds cannot be ruled out. It is known that beer contains phenolic compounds which are associated with positive impacts on health, and relatively low levels of ethanol. The physicochemical characterization of craft beers is also important for predicting the antioxidant activity of the final product. Although some studies exist regarding the chemical parameters, phenolic composition, and antioxidant capacities of beers, to the best of our knowledge, no studies regarding the determination of these parameters have been performed, in craft beers of different styles commonly consumed and found on the Portuguese market. Therefore, the study aims to determine their physicochemical parameters such as pH, total acidity, and reducing sugars; it also evaluates the total phenolic content (TPC) and antioxidant activity of selected Portuguese craft beers and raw materials used in their production, mainly hops and malt.

## 2. Results and Discussion

### 2.1. Physicochemical Analysis of Craft Beer Samples

The classification of beer in different styles is based on properties such as alcohol content, color, bitterness, clarity, flavor, and ingredients. There are several important parameters related to the quality of craft beer such as pH, total acidity, reducing sugars and total phenolic compounds. Table 1 shows the parameters analyzed in sample beers related to alcohol content, color, bitterness, pH, acidity and reducing sugar content. Beer is an alcohol source, although its content is variable depending on the type, ingredients, and fermentation. Beer ABV typically ranges from 3 to 14% when normal fermentation is used, but the most commonly consumed styles, do not exceed 6% [24]. According to Bamforth and Charles (2002), many beers have alcohol content ranging from 3% to 6% (*v/v*), including 56.2% of all the craft beers analyzed in our study [25] (Table 1). 

In this study, the alcohol content of craft beers varied between 4.5 (Pilsner) and 11.6 (Imperial Stout), while in industrial beers it ranges from 4.1 to 5.2% (Table 1). These results are in accordance with Bortoleto et al. (2022), who observed that the alcohol content in the industrial beer samples ranged from 4.5 to 5.3% (*v*/*v*), while in the craft samples the variation was from 4.4 to 9.1% (*v*/*v*), suggesting that the alcohol content tends to be higher in craft beer [26]. It is a fact that there is considerable variability in alcohol content within and across beverage type (e.g., beer, wine, and distilled spirits). For example, some light beers contain half as much alcohol as a regular beer, while some craft and specialty beers contain twice as much [27]. The higher values were seen in a specialty beer (Honey Beer—7.1) and in both Imperial Stout beers (10.0 and 11.6). The alcohol present in beers, apparently, can exert a neuroprotective effect, which can be linked to signal transduction activation processes potentially involving reactive oxygen species (ROS), several key protein kinases, and increased heat shock proteins [24]. Further, the presence of alcohol provides protection against several heart diseases and along with polyphenols can reduce oxidative stress [28].

As regards beer color, according to Baxter and Hughes (2001) the EBC scale ranges from 4.5 to 1550 and each color value will confer a beer flavor attribute [29]. Two Imperial Stout and one Oatmeal Stout beers showed the higher values of color, expressed in EBC (122, 130 and 123, respectively). These samples represent dark colored beers.

Bitterness is measured in IBUs which gives an approximate value of iso-α-acids present in milligrams of iso-α-acid per liter of beer [30]. Beer IBUs typically range between 5 and 120, and the popular use of higher quantities of more bitter hops in craft beers leads to higher IBU levels. In this study, bitterness values range from 13 (Munich Dunkel) to 80 (one India Pale Ale), expressed as IBU (Table 1). It has been shown that for beers, darker brown colors are associated with stronger, or more bitter, tastes/flavors [31]. This result is, in part, in accordance with our results which demonstrated that the two Imperial Stout beers showed high values for color and bitterness. Imperial Stout beers are also known for their medium to aggressively high bitterness [32]. Finally, it is important to note that the time of hop addition and hop variety used for beer production have been suggested as factors that may impact on bitterness quality, what can explain the range of values observed in this study [30]. Bitter beers, such as craft beers, tend to have a higher concentration of isohumulone, while industrial beers tend to have a higher concentration of isocohumulone [33]. 

In general, craft beers presented similar values of pH, ranging from 4.27 ± 0.03 to 4.92 ± 0.02. B-IPA (India Pale Ale beer) was the least acidic of all samples analyzed with a pH of 4.92, while AMP-VL (Vienna Lager beer) was the most acidic with a pH of 4.27. These results are in accordance with Granato et al. (2011), who reported pH values from 4.13 to 4.97 [34]. The total acidity of the beer samples ranged from 0.10 ± 0.00% to 0.62 ± 0.01% lactic acid equivalent (Table 1). The pH and total acidity are important criteria for brewers due to their influence on the sensory attributes, biological and chemical stability. In the case of light lager beers, the brewing industry usually prefers pH to be in the range of 3.90–4.20 [35].

Reducing sugar content in analyzed beers ranged from 2598.0 ± 0.0 to 4446.3 ± 31.1 mg/L glucose equivalents (Table 1). Considering that beers are usually presented in 0.33 L bottles, these results translate into a sugar content of 0.857 to 1.467 g per serving. As expected, these beers had a low sugar content, which are in accordance with other studies. For example, Pai et al. (2015) presented a reducing sugar content in the beer samples studied ranging from 0.469  ±  0.021 mg/mL to 2.682  ±  0.008 mg/mL [35]. The concentration of reducing sugar is an important parameter in the fermentation of beer because it provides information on optimization and regulation of the fermentation process to increase the yield and quality of the product [36]. Different contents of total and fermentable sugars are reported according to beer type. Yeast can only use certain lower molecular weight sugars, such as fructose, glucose, maltose, sucrose and maltotriose [35].

### 2.2. Total Phenolic Content and Antioxidant Activity of Beer Samples

In this study, 16 samples of craft beer and 3 samples of the most consumed industrial beers in the Portuguese market were analyzed regarding parameters related to antioxidant capacity (Table 2). So far more than 50 polyphenolic compounds have been identified in beer, of which 75% to 80% are derived from malt and 15% to 25% from hops [37]. These compounds improve the quality and acceptance of craft beers, influencing flavor and product stability, and contribute to the overall antioxidant activity of the beverage [34]. 

The main method to determine TPC is Folin–Ciocalteu assay. This is a colorimetric method based on electron transfer reactions between phenolic compounds and Folin–Ciocalteu reagent, resulting in blue color formation, proportional to the concentration of phenolic compounds [38]. 

In craft beers, TPC varied between 343.8 ± 22.2 GAE mg/L and 2172.5 ± 170.1 GAE mg/L (Table 2). In industrial beers, TPC varied between 255.3 ± 69.6 GAE mg/L and 394.0 ± 48.7 GAE mg/L. In craft beers, both Imperial Stout (AMP-IS, PS-IS) and one Oatmeal Stout (BG-OS) showed significantly higher values for TPC (2172.5 ± 170.0 GAE mg/L, 1749.3 ± 227.1 GAE mg/L and 1416.0 ± 80.4 GAE mg/L, respectively). The lowest values were observed in two industrial beers, both Pilsner (SB-S, 255.3 ± 69.6 GAE mg/L and SB-P, 312.6 ± 28.7 GAE mg/L). In their study, García-Guzmán et al. (2018) also observed that the highest polyphenol indices were obtained in stout beers [39].

Comparing our results with other studies, TPC values are higher in craft beers. For example, Piazzon et al. (2010) analyzed five different brands for each of the seven beer types and found different values depending on the beer type, ranging from 366 GAE mg/L for dealcoholized beers to 875 GAE mg/L for bock beers [40]. Marques et al. (2017) examined the TPC of four craft beers; values ranged from 448.57 to 531.30 GAE mg/L [41]. Granato et al. (2011) studied 29 beers (11 brown ale and 18 lager) and TPC ranged from 119.96 to 525.93 GAE mg/L, for laboratory produced beers [34]. Finally, Zhao et al. (2010) analyzed 34 commercial beer samples and found values varying from 152.01 GAE mg/L to 339.12 GAE mg/L [42]. These differences can be explained by beers with high original mash and with more dark/brown color, which tends to be associated with an increased value of phenolic compounds [38], such as craft beer samples BG-OS, AL-OS (Oatmeal stout), PS-IS, AMP-IS (Imperial stout), B-MD (Munich dunkel).

Our results also showed a value of TPC, for sample PS-HN (beer with honey), significantly higher than in industrial beers (950.4 ± 59.7 mg GAE/L). In vitro and in vivo studies have confirmed that honey possesses a range of antioxidant, antimicrobial, antiviral, anticancer, and antidiabetic properties [43,44]. Most of the biological activities of honey are attributed to its constituent phenolic and flavonoid compounds [45]. Nardini and Foddai (2020) reported that most special beers (six out of seven), including a beer with the addition of honey, showed TPC levels that were considerably and significantly (*p* < 0.05) higher (range 464–1026 mg/L of beer) as compared with those of conventional beers (range 274–446 mg/L of beer) [46].

The best TPC results observed in dark craft beers may be due to the special malts, such as crystal or caramel malts, and other coloring malts used in its production [47]. The TPC of craft beers is generally higher compared to industrial beers. This can be explained by large breweries often using less expensive raw materials and/or different techniques in the brewing process to produce a more cost-effective product. Craft breweries use raw materials such as barley and hops during beer production, which may be related (in part) to the presence of different and more abundant phenolic compounds in these beverages [13]. Moreover, craft beers are not submitted to filtration or pasteurization, processes that affect TPC [13,48]. 

Craft beers presented higher values of color, bitterness and antioxidant activity when compared to commercial beers [33]. Within the several stages that make up the beer production process, filtration emerges as one of the main factors responsible for the drastic reduction in the content of polyphenols present in the final matrix [49]. Consumers choose craft beers because they have a variety of flavors, such as malted barley, chestnut, and honey, which is related to a beverage of higher quality [50].

The DPPH method is widely used to assess antioxidant capacity. This method is based on the elimination of the stable free radical DPPH. The use of this radical has advantages: good stability in the absence of light, applicability, simplicity, viability, and the possibility of use in studies of antioxidant evaluation of pure substances, mixtures, or complex matrices [51].

Antioxidants with DPPH radical scavenging activity can donate hydrogen to free radicals, particularly to the lipid peroxides or hydroperoxide radicals that are the major propagators of the chain autoxidation of lipids, forming non-radical species, resulting in the inhibition of the propagating phase of lipid peroxidation. Beer with higher DPPH radical scavenging activity is important to flavor stability, which is the major determinant of the shelf life of this beverage, because beer staling is generally considered as the formation of saturated and unsaturated aldehydes, due to lipid oxidation [42].

Antioxidant capacity of craft beers was evaluated by measuring DPPH radical scavenging activity and metal chelating activity. For DPPH assay, the inhibition percentages varied between 58.3 ± 1.1% and 99.4 ± 0.6% (Table 2). These values are in accordance with those reported by Pai et al. (2015), who found values ranging from 68.34 ± 0.85% to 89.90 ± 0.71% [35]. On the other hand, the values obtained are higher than those reported by Granato et al. (2011), who reported values between 4.75 and 59.98% for Brazilian commercial beers [34], and Marques et al. (2017), who showed values ranging from 29.4% to 48.5% in craft beers (produced in the laboratory) [41].

Regarding craft beers, the sample AMP-IPA (India Pale Ale) showed the best value of DPPH radical inhibition (99.4 ± 0.6%) which was significantly higher than the industrial beers analyzed (*p* < 0.05). However, values for DPPH radical inhibition in craft and industrial beers were similar. In industrial beers, the higher value was 85.7 ± 2.1% (SB-P) and the lowest was 70.4 ± 0.7% (SB-S). Overall, the results regarding DPPH radical scavenging activity indicates a good beer stability and high antioxidant capacity. Samples with more DPPH radical scavenging do not necessarily present more TPC. Therefore, the amount of certain phenolic compounds rather than the quantity of TPC seems to determine the biological activity of beers with respect to antioxidant activity [52]. For example, phenolic acids strongly contribute to the antioxidant activity of beer [40], and flavonoids have been reported to be free radical scavengers, metal chelators, and strong antioxidants [53].

The metal chelating activity allows evaluation of the inhibition of the ferrozine-Fe^2+^ complex. Through the Fenton reaction (Fe^2+^ + H_2_O_2_ → Fe^3+^ + OH^−^ + OH^−^), the Fe^2+^ cation originates a hydroxyl radical. If the sample is able to chelate iron, it will prevent this reaction from happening, decreasing the production of free radicals [54]. Phenolic compounds in beer can act as chelating agents of metallic catalysts [55]. In this study, the metal chelating activity of samples varied from 1.6 ± 0.4% to 113.4 ± 15.8% (Table 2). The highest chelating activity was seen in sample AMP-IPA, an India Pale Ale (113.4 ± 15.8%), which also presented the highest DPPH scavenging activity and a good TPC value. Samples AMP-IS (Imperial Stout) and B-MD (Munich Dunkel) also presented values of metal chelating activity (46.2 ± 0.9% and 67.0 ± 1.2%, respectively) significantly higher than industrial beers (*p* < 0.05). 

Other authors also evaluated metal chelating activity in beer samples. Zhao et al. (2010) presented values ranging from 0.12 µmol EDTA Equivalents (EDATE)/L to 54.57 µmol EDTAE/L and reported that the raw materials and the brewing process can have a major impact on the metal chelating activity [42]. 

In this study, beer color has a positive, statistically significant correlation with TPC (Table 3). This result was expected, since it is known that darker beers have a higher content of polyphenols [14]. Alcohol content and TPC also have a positive, statistically significant correlation. The effect of ethanol content might be explained by the higher solubility of phenolic compounds in this solvent in comparison to water, increasing their extraction from raw materials during brewing [56]. Higher phenolic compounds in dark beers along with higher alcohol content is due to the fact that these beers are brewed from wort with higher extract content. Moreover, beers with higher original gravity generally showed higher TPC (>180 mg GAE/L), indicating that phenolic compounds in beer mainly originated from barley malt and hops, although the brewing process itself may influence the final polyphenols content and antioxidant activity of the beers [55]. Gorjanović et al. (2010) also reported that polyphenols content is lower in alcohol-free beers because they are usually brewed with lower original wort extract and inhibition of alcohol formation [57]. 

The correlation between parameters related to antioxidant activity (DPPH scavenging activity and metal chelating activity) stands out, suggesting that overall antioxidant activity evaluation results were consistent although these assays involved different reaction mechanisms. Therefore, compounds that can inhibit DPPH radicals are capable of chelating ferrous ions. A lack of correlation between TPC and the antioxidant activity methods was reported by other authors [42,58]. 

Significant positive correlations between alcohol content, color, or bitterness and TPC were observed. Beer labels contain information of their alcohol content, and in some samples, they also included color indication (EBC) and/or bitterness (IBU). The presence of this information on the label can be important for the consumer, helping to choose a product with a greater presence of phenolic compounds and a beverage with increased health value. A significant positive correlation between bitterness and metal chelating activity was also found, reinforcing the importance of indicating bitterness on labels.

### 2.3. Antioxidant Activity of Raw Materials

The antioxidant activity of the raw materials, namely malt and hops, used in the production of craft beers was also analyzed. The malt and hop samples used in this study were analyzed as pure ingredients and not as the specific mixtures actually used to brew specific beers, and this is because the recipe was confidential, preventing reproduction of the mixture.

Malted barley is the second highest ingredient, in proportion, after water in brewing. Barley contains high levels of β-glucans and phenolic compounds with antioxidant properties. During malting, the extractability of phenolic compounds increases mainly due to enzymatic processes and better friability [59]. Malts are not all equal and their chemical composition largely depends on the time and temperatures of the production process [60]. Specialty malts are produced not for their enzyme content but to provide extra color and flavor to beer [61]. 

For malt, DPPH scavenging activity varied between 36.5 ± 2.8% and 96.0 ± 2.1% (Table 4). These results are similar to those observed by Coghe et al. (2005). In their study, the authors reported 16% to 89% of radical scavenging activity for wort samples [62]. In contrast, Koren et al. (2019) found values above 80% for all their malt samples; however, malt extraction was performed with a solution of 80% acetone and 20% water. The use of different extraction solutions (water vs. acetone and water solution) may influence the extracted compounds, explaining the difference in the results [59]. Antioxidants from malt are able to scavenge oxygen free radicals and prevent oxidative reactions, avoiding the addition of exogenous antioxidant [63]. Different phenolic compounds have been identified in barley and malt, including flavan-3-ols, proanthocyanidin oligomers, hydroxycinnamic acid derivatives, and flavonols [61], which may be related to the antioxidant activity observed in our samples, regarding DPPH assay. 

Barley and wheat are the most common grains used in brewing. During the process, the grain is malted, milled, and mashed to convert starch to sugar, to be used for fermentation [5]. In the present study, 9 of the 10 malts were barley malts and only one was a wheat malt. The highest value was seen in Pale Ale malt (96.0%), which corresponds to a barley malt, and the lowest value was observed for wheat malt (36.5%). In their study, Fogarasi et al. (2015) tested different cereals, namely, one organic einkorn wheat (*Triticum monococcum* L.), one organic barley (*Hordeum vulgare* L.), and eight bread wheat (*T. aestivum*) varieties. In all cases the barley sample had the highest antioxidant potential and polyphenol content. Values for DPPH scavenging activity observed in two base malts (wheat malt, 36.5 ± 2.8% and Pils malt, 47.5 ± 5.8%) were significantly lower (*p* < 0.05) than those observed in all the other malts. Base malts are used mainly to add fermentable sugars to the beers instead of adding aroma, flavor, and color [64]. 

Metal chelating activity in malt samples varied between 12.0 ± 0.5% and 24.8 ± 0.6%, with significantly higher value corresponding to Munich malt (24.8 ± 0.6%), *p* < 0.05. Munich-style barley malt is known to exhibit antioxidant properties that are beneficial in stabilizing beer flavor [65]. The existence of metal chelating activity can be explained by the presence of possible chelating agents, such as some phenolic compounds that can inhibit radical generation by stabilizing transition metals, and consequently reducing free radical damage [66]. 

The presence of hops (*Humulus lupulus* L.) in beer production has a crucial impact on beer quality. The brewing industry uses various hops varieties differing in content and composition of bioactive compounds, which can be associated with differences in antioxidant properties [67]. The importance of hops polyphenols in the brewing process is due to protein-polyphenol interaction of nonbiological haze, which limits the shelf life of bottled beers [68]. Hop cones contain polyphenols such as multifidol glucosides and prenylflavonoids (e.g., xanthohumol, desmethylxanthohumol, 6-prenylnaringenin, and 8-prenylnaringenin) [69]. 

In this study, hop samples were prepared to simulate the brewing process. In DPPH scavenging activity assay, results ranged from 64.7% (Magnum variety) to 79.6% (organic sample), all values above 50% of inhibition (Table 5). Mudura et al. (2010) reported values between 3.54% and 13.45% for DPPH scavenging activity for hops cultivated in Romania [70]. The differences observed in these studies may be explained by the fact that the antioxidant content of hops can be influenced, for example, by soil and weather conditions during vegetation and ripening, hop plant age and harvest time, geographic and/or cultivar differences [71]. 

The results showed that bittering hops had the lowest scavenging activity (significantly lower inhibition percentage—64.7 ± 6.5%, *p* < 0.05), when compared with the majority of aromatic and dual-purpose hops. The results are consistent with findings published by Mudura et al. (2010), where aromatic varieties showed higher content of polyphenolic compounds and higher anti-radical activity (Huller Bitterer, 13.45%), when compared with bittering hops (e.g., Magnum, 3.54%) [70].

For metal chelating activity, and using water as solvent, the results varied between 3.8 ± 0.5% (Mittelfruh hop) to 23.5 ± 2.4% (Hersbrucker hop). Hersbrucker, an aromatic hop, was the sample with the highest value of metal chelating activity (significantly higher inhibition percentage—23.5 ± 2.4%). In their study, Kobus-Cisowska et al. (2019) reported higher values for metal chelating ability in samples, above 20% in water extracts. However, a different methodology in sample preparation was employed, since a three-step extraction method was used and the extracts were centrifuged [72]. Another possible explanation is that the hops used in both studies may have different origins and cultivation conditions. In their study, Kobus-Cisowska et al. (2019) showed that among the analyzed hop cone extracts, the highest amount of iron ions (55.43–88.76%) was chelated by the ethanol extracts of the Magnum cultivar, and lower metal chelating ability was demonstrated for water extracts [72]. 

Comparing the results from DPPH assay in malt and hop samples, values were higher in malt samples (excluding two base malts). It is known that around 80% of the phenolic compounds identified in beer is derived from malt, while the remaining 20% comes from hop [15,73]. Moreover, malt can contribute to about 95% and 86% of the antioxidant capacity of dark and pale beers, respectively [74].

Finally, comparing the results obtained for raw materials (malts and hops) with craft beers, beers have a significantly higher DPPH scavenging activity and metal chelating activity (both with *p* = 0.05). Two possible explanations are a synergistic effect of phenolic compounds present in raw materials and the increased solubility of these compounds in hydroalcoholic solutions [14]. 

## 3. Material and Methods

### 3.1. Chemicals 

Sodium hydroxide (NaOH), phenolphthalein and Folin–Ciocalteu reagent, dimethyl sulfoxide (DMSO), and iron (II) sulphate heptahydrate, were purchased from VWR (Portugal). Potassium sodium tartrate tetrahydrate, 3,5-dinitrosalicylic acid (DNS), glucose, sodium carbonate, gallic acid, absolute ethanol, 2,2-diphenyl-1-picrylhydrazyl (DPPH), ferrozine and ethylenediamine tetraacetic acid (EDTA) were purchased from Sigma-Aldrich (USA). 

### 3.2. Beer Samples and Raw Materials

A total of 16 Portuguese craft beers were purchased in supermarkets (Porto, Portugal) and obtained from the donation of craft breweries (Porto and Lisbon craft breweries). Three industrial beers, used for comparison purposes, were also collected in supermarkets. The brand names were omitted and represented by letters codes, as summarized in Table 6, together with the characteristics and packaging specifications of all beers. During the study, together with craft beers, the starting malts (10 samples) and hops (11 samples), from different varieties, were obtained from Portuguese craft breweries. These raw materials were used in the production of the craft beers analyzed. 

### 3.3. Sample Preparation

For craft beers, the content of each bottle was homogenized (stirring with a glass rod for 10 s). Then, samples were degassed by sonication (Sonorex Super RK 100/H, Bandelin, Germany) for 40 min at 35 kHz, and room temperature. The disappearance of the bubbles was indicative of the absence of CO_2_ [34,75]. Beer bottles were stored in the dark and analyzed immediately after opening. Aliquots were frozen at −80 °C until analysis. 

Malt samples were prepared according to Mareček et al. (2017). Briefly, the malt was ground in an electric mill and 25 g of the sample was weighed. Then, 225 mL of distilled water was added to the malt and placed in a 45 °C water bath for 15 min. After cooling, it was filtered and kept at −20 °C until use [76].

Hop samples were prepared according to Krofta et al. (2008), with minor modifications. The hop pellets were ground with an electric mill and 5 g of dry hop material was weighed out. The ground hops were transferred to an Erlenmayer with boiling water and allowed to boil for 30 min. After cooling, the flask content was transferred to a 1000 mL volumetric flask and the volume was completed with distilled water. The extract was filtered and kept at −20 °C until use [67]. 

### 3.4. Chemical Analysis of Craft Beer Samples

pH, total acidity, reducing sugar content, and TPC were measured in all beer samples. Craft breweries provided data regarding beer color (expressed in European Brewing Convention (EBC) units), bitterness (expressed as International Bittering Units (IBU)) and alcohol content (by volume (ABV)) (Table 1).

### 3.5. pH Determination

The pH was measured in 100 mL of the degassed beer and using a calibrated pH meter (Symphony, VWR, Lisbon, Portugal). 

### 3.6. Total Acidity (TA)

The acidity was measured by titration (50 mL of degassed beer) with a 0.1 M NaOH solution in the presence of phenolphthalein as the indicator until the appearance of a pale pink color that persisted for 1 min [75]. In the darkest samples, titration was performed with a calibrated pH meter, measuring the amount of sodium hydroxide required to raise the pH to 8.2. Total acidity of beer was calculated using the formula: $TA\;\left(as\;Lactic\;Acid\right)=\frac{V\times 0.9}{50}$, where *V* = volume in mL of 0.1 M NaOH used [77,78]. 

### 3.7. Reducing Sugar Content

The analysis of reducing sugar content was carried out using the DNS method, proposed by Başkan et al. (2016), with minor modifications. Briefly, a solution of DNS (1%) was prepared by dissolving 1 g of DNS in 20 mL of NaOH (2 M). After that, 30 g of sodium and potassium tartrate were added, and the mixture diluted with distilled water (1 L). Then, 500 µL of beer sample was mixed with 500 µL of the DNS solution and vortexed vigorously. The mixture was incubated in a water bath at 100 °C, for 5 min. As a standard solution, different concentrations of glucose solution were used (50, 100, 200, 400, 600, 800 and 1000 mg/L). The absorbance was read at 540 nm in a UV-Vis spectrophotometer (Model VWR UV-1600PC). With the data obtained, a linear regression line was calculated using the standard glucose solutions. Results were expressed in glucose equivalents (GE)/100 mL of beer sample [22].

### 3.8. Parameters Related to Antioxidant Capacity

#### 3.8.1. Total Phenolic Content (TPC)

TPC was determined according to the spectrophotometric method Folin–Ciocalteu, described by Marques et al., (2017). Briefly, 1.25 mL of Folin–Ciocalteu reagent (0.2 M) was added to 250 µL of degassed beer sample or gallic acid GA (standard solution) and allowed to stand for 5 min. Then, 2 mL of sodium carbonate (Na_2_CO_3_) solution (75 g/L) was added. Distilled water was added until 5 mL. The solution was incubated for 1 h at room temperature in the dark and then the absorbance was read at 760 nm using a UV-Vis spectrophotometer (Model VWR UV-1600PC). Absorbance values were converted to gallic acid equivalents (GAE) mg/L craft beer through a calibration curve obtained with standard GA in water [41]. 

#### 3.8.2. DPPH Scavenging Activity

For DPPH scavenging activity, in a 96-well plate, a 19.4 µL aliquot of sample was added to 175 µL of DPPH (100 µM) radical, and the absorbance was measured at 517 nm, using a microplate reader (Model MR133T, DYnex). Readings were repeated every 5 min, until the end of the reaction. DPPH scavenging activity was calculated using the following formula: $Inhibition\left(\%\right)=\frac{\left(AC-AS\right)}{AC}\times 100$, where AC is the absorbance of the control and AS represents the absorbance of the sample [79].

#### 3.8.3. Metal Chelating Activity (MCA)

Measurement of metal chelating activity or Ferrozine assay was carried out according to the methodology described by Russo et al. (2005). Briefly, a 96-well plate was prepared by adding 50 µL of sample or EDTA (positive control) and 50 µL of 0.15 mM ferrous sulfate solution (FeSO4) to each well. The plate was left to stand for 5 min and then 50 µL of 0.5 mM ferrozine was added to each well. The mixture was vigorously stirred and left for 10 min at room temperature and protected from light. Absorbance was measured at 562 nm using a microplate reader (Model MR133T, DYnex). With the data obtained, the chelating capacity of the samples was calculated using the following formula: $Chelating\;Activity\left(\%\right)=\frac{\left(AC-AS\right)}{AC}\times 100$, where AC is the absorbance of the control and AS represents the absorbance of the sample [80].

### 3.9. Statistical Analysis

Data are presented as the mean ± standard deviation (SD) for triplicate determinations, and samples were collected from the same production lot. The results were assessed through statistical analysis of simple variance (one-way ANOVA), to detect statistically significant differences, using IBM^®^ SPSS^®^ Statistics (version 26.0, sourced from Porto, Portugal). The Tukey test was also applied to identify samples with significant differences between them (95% significance). Correlation coefficients (r) were calculated using Pearson Product Moment Correlation, to determine the correlations among means. Differences with a *p* < 0.05 were considered significant.

## 4. Conclusions

The present study describes the variations in chemical parameters, phenolic content and antioxidant activities of selected Portuguese craft beers and raw materials used in their production. There were considerable variations in total phenolic content and antioxidant activities of beers across different samples. The phenolic content of a beer depends on several factors such as the differences in raw materials and brewing process. Nonetheless, several beers showed a high content of phenolic compounds and good antioxidant activity. However, there was a lack of correlation between TPC and the antioxidant activity (DPPH and MCA). Commercial beer exhibited a lower phenolic content than craft beers. It was observed that many beer characterization parameters (such as alcohol content, color, bitterness, total acidity or reducing sugars content) were correlated with TPC, which can be used as an indicator in consumer choice. Beer samples revealed higher antioxidant capacity than the raw materials. Raw materials showed different antioxidant activities, indicating that a careful selection is needed to obtain a final product with higher antioxidant capacity. This work allowed measurement of the antioxidant capacity of the analyzed beers and the influence of the raw materials in this parameter. It would be relevant to extend this study to more craft beers available on the market, produced in Portugal, and to analyze individual phenolic contents.

## Figures and Tables

**Table 1:** Principal chemical parameters of each analyzed beer.

Beer Sample	Beer Style	Alcohol (ABV%)	Color (EBC)	Bitterness (IBU)	pH	Total Acidity (%)	Sugar (mg/L)
BG-SB	Strong Bitter	5.2	17	40	4.43 ± 0.02	0.18 ± 0.00	2598.0 ± 0.0
BG-OS	Oatmeal Stout	6.5	123	42	4.55 ± 0.01	0.51 ± 0.02	2598.0 ± 0.0
PS-P	Pilsner	4.5	7	36	4.81 ± 0.01	0.15 ± 0.00	2598.0 ± 0.0
PS-IS	Imperial Stout	11.6	130	60	4.37 ± 0.03	0.62 ± 0.01	3844.2 ± 438.8
PS-HN	Special (Honey Beer)	7.1	37	26	4.28 ± 0.04	0.32 ± 0.01	2598.0 ± 0.0
N-LAG	Lager	5.0	12	18	4.57 ± 0.00	0.18 ± 0.01	2598.0 ± 0.0
N-IPA	India Pale Ale	6.0	15	50	4.61 ± 0.01	0.23 ± 0.00	4070.5 ± 23.8
B-MD	Munich Dunkel	5.2	40	20	4.78 ± 0.05	0.37 ± 0.00	3077.2 ± 94.1
B-BA	Blond Ale	4.8	10	13	4.43 ± 0.03	0.17 ± 0.00	2239.7 ± 62.6
B-IPA	India Pale Ale	6.5	17	40	4.92 ± 0.02	0.28 ± 0.01	3798.0 ± 39.3
AL-W	Witbier	5.0	8	17	4.46 ± 0.01	0.17 ± 0.00	2598.0 ± 0.0
AL-OS	Oatmeal Stout	5.5	88	39	4.49 ± 0.01	0.44 ± 0.01	4406.3 ± 25.2
AL-IPA	India Pale Ale	6.5	23	55	4.69 ± 0.03	0.33 ± 0.00	4294.2 ± 28.8
AMP-IS	Imperial Stout	10.0	122	67	4.77 ± 0.02	0.48 ± 0.02	4446.3 ± 31.1
AMP-VL	Vienna Lager	5.4	21	25	4.27 ± 0.03	0.27 ± 0.00	2598.0 ± 0.0
AMP-IPA	India Pale Ale	7.0	21	80	4.84 ± 0.04	0.44 ± 0.02	4049.7 ± 18.8
SB-P *	Pilsner	5.2	8	16	4.50 ± 0.07	0.15 ± 0.00	2598.0 ± 0.0
SB-S *	Pilsner	5.0	6	30	4.45 ± 0.06	0.11 ± 0.00	2598.0 ± 0.0
SG-P *	Munich Dunkel	4.1	39	13	4.56 ± 0.05	0.10 ± 0.00	2598.0 ± 0.0

* Industrial beers.

**Table 2:** Phenolic content, DPPH radical scavenging activity and metal chelating activity in analyzed beers.

Beer Sample	Beer Style	TPC (mg GAE/L)	DPPH (% Inhibition)	Metal Chelating Activity (%)
BG-SB	Strong Bitter	555.1 ± 49.4 ^b^	58.3 ± 1.1 ^a^	14.1 ± 1.1
BG-OS	Oatmeal Stout	1416.0 ± 80.4 ^a,b,c^	ND	30.6 ± 0.7 ^a^
PS-P	Pilsner	343.8 ± 22.2	87.8 ± 5.0 ^b^	22.2 ± 1.9 ^a^
PS-IS	Imperial Stout	1749.3 ± 227.1 ^a,b,c^	82.2 ± 4.4	3.4 ± 0.5 ^b,c^
PS-HN	Special (Honey Beer)	950.4 ± 59.7 ^a,b,c^	96.8 ± 8.2 ^b^	1.6 ± 0.4 ^b,c^
N-LAG	Lager	484.5 ± 40.1	73.4 ± 4.6 ^a^	21.7 ± 0.2 ^a^
N-IPA	India Pale Ale	513.8 ± 26.5 ^b^	76.7 ± 2.8	27.6 ± 0.3 ^a^
B-MD	Munich Dunkel	752.2 ± 83.3 ^a,b,c^	ND	67.0 ± 1.2 ^a,b,c^
B-BA	Blond Ale	449.9 ± 18.0	80.1 ± 6.7	22.9 ± 1.4 ^a^
B-IPA	Indian Pale Ale	588.9 ± 70.0 ^a,b^	95.8 ± 3.4 ^b^	17.0 ± 0.7
AL-W	Witbier	410.7 ± 37.4	95.4 ± 3.0 ^b^	22.8 ± 1.3 ^a^
AL-OS	Oatmeal Stout	757.8 ± 30.1 ^a,b,c^	97.9 ± 1.3 ^b^	23.5 ± 2.4 ^a^
AL-IPA	India Pale Ale	758.0 ± 89.1 ^a,b,c^	59.5 ± 3.8 ^a^	8.6 ± 0.6 ^b,c^
AMP-IS	Imperial Stout	2172.5 ± 170.1 ^a,b,c^	ND	46.2 ± 0.9 ^a,b,c^
AMP-VL	Vienna Lager	658.4 ± 23.4 ^a,b,c^	88.8 ± 7.0 ^b^	12.5 ± 0.7
AMP-IPA	India Pale Ale	936.4 ± 53.1 ^a,b,c^	99.4 ± 0.6 ^a,b^	113.4 ± 15.8 ^a,b,c^
SB-P	Pilsner	312.6 ± 28.7	85.7 ± 2.1 ^b^	7.6 ± 0.3 ^b,c^
SG-S	Pilsner	255.3 ± 69.6	70.4 ± 0.7	23.1 ± 1.3 ^a^
SG-P	Munich Dunkel	394.0 ± 48.7	ND	21.3 ± 0.5 ^a^

ND—Not determined. Values are means ± SD (*n* = 3). ^a^ Significantly different compared with SB-P (*p* < 0.05); ^b^ Significantly different compared with SG-S (*p* < 0.05); ^c^ Significantly different compared with SG-P (*p* < 0.05) (ANOVA followed by post-tests).

**Table 3:** Correlations among beer parameters, antioxidant activity evaluation indices and total phenolic contents.

	TPC	ABV	EBC	IBU	PH	RS	TA	DPPH	MCA
TPC	1	0.880 **	0.868 **	0.628 **	0.069	0.407 **	0.858 **	0.154	0.168
ABV	-	1	0.719 **	0.691 **	0.023	0.457 **	0.801 **	0.107	0.036
EBC	-	-	1	0.446 **	−0.026	0.326 *	0.847 **	0.144	0.034
IBU	-	-	-	1	0.406 **	0.734 **	0.659 **	0.013	0.440 **
PH	-	-	-	-	1	0.501 **	0.110	0.120	0.552 **
RS	-	-	-	-	-	1	0.518 **	0.127	0.356 **
TA	-	-	-	-	-	-	1	0.289	0.262
DPPH	-	-	-	-	-	-	-	1	0.302 *
MCA	-	-	-	-	-	-	-	-	1

ABV, alcohol by volume; IBU, bitterness; EBC, color; RS, Reducing Sugars Content; TA, Total Acidity; TPC, Total Phenolic Content; DPPH, DPPH radical scavenging activity; MCA, Metal Chelating Activity. * Correlation is significant at the 0.05 level (2-tailed); ** correlation is significant at the 0.01 level (2-tailed).

**Table 4:** Antioxidant Activity of Malt Samples.

Sample	Type of Malt	DPPH (% Inhibition)	Metal Chelating Activity (%)
Munich	Base	92.5 ± 7.2 ^b^	24.8 ± 0.6 ^d^
Pils	Base	47.5 ± 5.8 ^a^	19.8 ± 1.7 ^c^
Wheat	Base	36.5 ± 2.8 ^a^	13.5 ± 1.9 ^a^
Pale Ale	Base	96.0 ± 2.1 ^b^	20.3 ± 0.1 ^c^
Biscuit	Specialty	90.7 ± 2.2 ^b^	18.8 ± 2.8 ^b,c^
Carapils	Specialty	85.1 ±3.5 ^b^	20.5 ± 0.7 ^c^
Crystal Light	Specialty	90.5 ±5.4 ^b^	14.9 ± 1.0 ^a,b^
Chocolate	Specialty	ND	14.2 ± 0.6 ^a^
Chateau Special	Specialty	ND	19.0 ± 1.5 ^c^
Cara Ruby	Specialty	ND	12.0 ± 0.5 ^a^

ND—Not determined. Values are means ± SD (*n* = 3). Means with different superscript letters in the same column are significant differences (*p* < 0.05, ANOVA followed by post-tests).

**Table 5:** Antioxidant Activity of Hops Samples.

Hop Varieties	Brewing Use	DPPH (% Inhibition)	Metal Chelating Activity (%)
Organic	-	79.6 ± 2.6 ^b^	16.2 ± 3.9 ^b,c^
Magnum	Bittering	64.7 ± 6.5 ^a^	14.2 ± 1.7 ^b,c^
Mittelfruh	Aromatic	76.3 ± 3.3 ^b^	3.8 ± 0.5 ^a^
Hersbrucker	Aromatic	78.2 ± 1.0 ^b^	23.5 ± 2.4 ^d^
Celeia	Aromatic	77.9 ± 2.6 ^b^	11.9 ± 4.0 ^b^
East Kent	Aromatic	73.8 ± 1.8 ^a,b^	12.1 ± 0.3 ^b^
Citra	Dual Purpose	76.4 ± 2.4 ^b^	18.3 ± 1.6 ^c,d^
Mosaic	Dual Purpose	78.3 ± 0.5 ^b^	12.3 ± 1.1 ^b,c^
Simcoe	Dual Purpose	78.0 ± 5.4 ^b^	16.2 ± 0.7 ^b,c^
Centennial	Dual Purpose	76.8 ± 1.7 ^b^	15.4 ± 1.9 ^b,c^
Perle	Dual Purpose	76.5 ± 2.6 ^b^	14.7 ± 0.2 ^b,c^

Values are means ± SD (*n* = 3). Means with different superscript letters in the same column are significant differences (*p* < 0.05, ANOVA followed by post-tests).

**Table 6:** Characteristics of each beer sample regarding its production, packaging type and source.

Beer Samples	Craft vs. Industrial	Beer Style	Beer Color	Packing	Packing Volume (mL)	Source
BG-SB	Craft Beer	Strong Bitter	Pale Amber	Bottle	330	Brewery
BG-OS	Craft Beer	Oatmeal Stout	Black	Bottle	330	Brewery
PS-P	Craft Beer	Pilsner	Deep Gold	Bottle	330	Brewery
PS-IS	Craft Beer	Imperial Stout	Black	Bottle	330	Brewery
PS-HN	Craft Beer	Special (Honey Beer)	Amber Brown	Bottle	330	Brewery
N-LAG	Craft Beer	Lager	Deep Gold	Bottle	330	Brewery
N-IPA	Craft Beer	India Pale Ale	Pale Amber	Bottle	330	Brewery
B-MD	Craft Beer	Munich Dunkel	Brown	Bottle	330	Brewery
B-BA	Craft Beer	Blond Ale	Pale Gold	Bottle	330	Brewery
B-IPA	Craft Beer	Indian Pale Ale	Pale Amber	Bottle	330	Brewery
AL-W	Craft Beer	Witbier *	Pale Gold	Bottle	330	Brewery
AL-OS	Craft Beer	Oatmeal Stout **	Black	Bottle	330	Brewery
AL-IPA	Craft Beer	India Pale Ale	Medium Amber	Bottle	330	Brewery
AMP-IS	Craft Beer	Imperial Stout	Black	Bottle	330	Brewery
AMP-VL	Craft Beer	Vienna Lager	Pale Amber	Bottle	330	Brewery
AMP-IPA	Craft Beer	India Pale Ale	Pale Amber	Bottle	330	Brewery
SB-P	Industrial Beer	Pilsner	Pale Gold	Bottle	250	Supermarket
SB-S	Industrial Beer	Pilsner	Straw	Bottle	250	Supermarket
SG-P	Industrial Beer	Munich Dunkel	Brown	Bottle	250	Supermarket

* With honey, coriander, orange peel and pennyroyal; ** with carob and fig.

## Data Availability

Not applicable.

## Sample Availability

Samples are not available from the authors.
